# Effect of sodium hypochlorite irrigant temperature on postoperative pain following endodontic treatment: a systematic review

**DOI:** 10.3389/fdmed.2026.1793274

**Published:** 2026-04-23

**Authors:** Maria Anna Geevarghis, Chitharanjan Shetty, Teena Sheethal Dsouza, Shreya Hegde, Derek Shaji Pious, Rashi Shroff

**Affiliations:** 1 Nitte (Deemed to be University), AB Shetty Memorial Institute of Dental Sciences (ABSMIDS), Department of Conservative Dentistry & Endodontics, Mangalore, India; 2 Department of Conservative Dentistry and Endodontics, Manipal College of Dental Sciences Mangalore, Manipal Academy of Higher Education, Manipal, India

**Keywords:** cryotherapy, endodontics, irrigant temperature, postoperative pain, randomized controlled trials, sodium hypochlorite

## Abstract

**Background:**

This systematic review thoroughly evaluates the impact of various temperatures of sodium hypochlorite (NaOCl) used during root canal therapy (RCT) on postoperative pain (POP).

**Methods:**

After registering with PROSPERO (CRD420251235909, https://www.crd.york.ac.uk/PROSPERO/view/CRD420251235909), a search was carried out by using PubMed, Scopus, Web of Science, Cochrane library published in the years 2015 January 1 to 2025 November 16. Randomized clinical trials comparing NaOCl irrigant temperatures and reporting POP outcomes were included.

**Results:**

Three randomised Control Trial (total N ≈ 183 patients) assessed NaOCl temperature (cold ≈ 2–2.5 °C, room ≈ 22–25 °C, warm ≈ 40–66 °C). Two trials reported lower postoperative pain with cold NaOCl compared with warmer solutions at early timepoints (notably 6 h or day-1). Another trial (various concentrations and temperatures) found no statistically significant difference across temperatures or concentrations at the timepoints measured. Heterogeneity in diagnosis (vital vs. non-vital teeth), irrigant concentration, scale used for pain measurement (0–10 vs. 0–100), and timepoints limited performing meta-analysis.

**Conclusions:**

Evidence from recent RCTs suggests a short-term reduction in early postoperative pain with cold NaOCl vs. warm NaOCl or room temperature in some settings, while other trials show no difference. Temperature modulation is promising but not conclusively proven; further standardized RCTs (harmonized pain scales, same timepoints, same diagnoses) or pooled analyses with harmonized data are needed.

**Systematic Review Registration:**

https://www.crd.york.ac.uk/PROSPERO/view/CRD420251235909, PROSPERO CRD420251235909.

## Introduction

There are incidences of post-operative pain (POP) ranging between 3% and 58% following root canal treatment (RCT) depending on the clinical, biological and procedural variables. It is one among the common and distressing complications for these patients (1, 2). Although POP being multifactorial, the quality of chemomechanical debridement plays a crucial role in limiting apical extrusion of debris, reducing inflammatory responses, and improving patient-centered outcomes. The microorganisms located in anatomical complexities such as fins, isthmuses, and lateral canals cannot be eliminated by mechanical instrumentation alone (3, 4). Therefore, the usefulness of irrigation protocols becomes very important.

Broad-spectrum antimicrobial action and superior organic tissue dissolution ability selects sodium hypochlorite (NaOCl) to be widely used as an endodontic irrigant (5, 6). Despite these benefits, NaOCl is extremely reactive and can cause irritation of periapical tissues, when extruded or used at higher concentrations (7). Various approaches such as changing concentration, volume, agitation methods and more recently temperature modification have been explored to optimize its antimicrobial and tissue-dissolving properties while minimizing adverse effects.

Evidence suggests that NaOCl's physicochemical behavior can be greatly affected by variations in temperature. Heating NaOCl increases its availability of chlorine, promotes the dissolution of organic tissue, and may enhance the process of elimination of smear layers (8). However, methodological heterogeneity has hindered the establishment of clear clinical recommendations, and outcomes from these trials are still inconsistent.

There is a strong need for collecting the available data given the growing interest in irrigant temperature modulation and the lack of a previous systematic synthesis that focused only on temperature variation of NaOCl. Determining whether temperature-controlled NaOCl irrigation can lessen pain following procedure has direct clinical implications for enhancing patient comfort and maximizing endodontic results.

Therefore, this systematic review's goal is to assess how the temperature of the sodium hypochlorite irrigant—cold, room temperature, or warm—affects POP in human permanent teeth after nonsurgical endodontic treatment.

## Methodology

This systematic review was completed and stated in accordance with the Preferred Reporting Items for Systematic Reviews and Meta-Analyses (PRISMA 2020) guidelines, and the protocol was previously registered in PROSPERO (Registration ID: CRD420251235909, https://www.crd.york.ac.uk/PROSPERO/view/CRD420251235909).

### Research question

Following the Population–Intervention–Comparator–Outcome–Study Design (PICOS) format, the question guiding this review was: “Does irrigation with NaOCl at different temperatures—cold, room temperature, or warm (I)—as opposed to standard room-temperature NaOCl (C) affect POP intensity (O) in patients undergoing non-surgical root canal treatment (P) in randomized controlled trials (S)?” Assessing if analgesic consumption varied between temperature-modulated NaOCl groups was the secondary goal.

### Eligibility criteria

Study selection was conducted according to predefined criteria structured around the Population, Intervention, Comparator, Outcome, and Study Design (PICOS) framework. The population consisted of human participants undergoing nonsurgical root canal treatment in permanent teeth, including both single-rooted and multi-rooted teeth, diagnosed with symptomatic irreversible pulpitis or symptomatic or asymptomatic apical periodontitis. The intervention of interest involved irrigation with cold or precooled sodium hypochlorite (NaOCl) maintained at temperatures between 0 and 4 °C, either during instrumentation or as the final irrigating solution. These interventions were compared with the use of NaOCl at room temperature (20–25 °C) or warmed/heated NaOCl (≥40 °C). Studies evaluating two or more NaOCl temperature conditions were considered eligible for inclusion. The primary outcome assessed was postoperative pain, measured using validated pain assessment instruments such as the Visual Analog Scale (VAS) or the Heft–Parker Visual Analog Scale (HP-VAS). Analgesic intake following treatment was considered as a secondary outcome. Only randomized controlled trials, including both parallel-arm and split-mouth designs, performed as single-visit or multiple-visit endodontic procedures and published in peer-reviewed journals, were included. Furthermore, eligible studies were required to report essential procedural details such as working length determination, instrumentation technique, apical preparation size, irrigation protocol, and obturation method to allow meaningful comparison of postoperative pain outcomes.

Studies were excluded if they were conducted on animals or represented *in vitro*, *ex vivo*, or simulation models. Investigations involving primary teeth, retreatment cases unless specifically defined within the protocol, or teeth with open apices, root resorption, traumatic injuries, or cystic lesions were also excluded. Studies that evaluated irrigants other than NaOCl or failed to control or compare irrigant temperature were not considered eligible. Similarly, trials focusing exclusively on variations in NaOCl concentration, irrigation volume, or activation techniques without temperature modification were excluded. Studies that did not report postoperative pain outcomes, used non-validated pain assessment tools, or assessed only antibacterial effects without patient-reported pain outcomes were also excluded. Additionally, non-randomized studies, observational designs, case reports, case series, narrative reviews, systematic reviews, meta-analyses, and conference abstracts lacking full-text availability were excluded from the analysis.

### Information sources and search strategy

An extensive electronic search was undertaken over four main bibliographic databases—PubMed, Scopus, Web of Science and Cochrane library—to recognise relevant randomized controlled trials evaluating the consequence of NaOCl irrigant temperature on POP following nonsurgical root canal treatment. Searches were restricted to articles published between 2015 and 2025 to analyse the most contemporary evidence. Earlier studies did not specifically investigate irrigant temperature as an independent variable, or differed substantially in methodology and outcome reporting, which would reduce comparability with more recent outcomes.

The search strategy (Table 1) was improved using combinations of Medical Subject Headings (MeSH) and free-text terms linked to postoperative endodontic pain, sodium hypochlorite, and irrigant temperature modification. Search terms were formed using Boolean operators:

**Table 1:** Database and search string.

Database	Search string	Results
PUBMED	((“postoperative pain"[tiab] OR “postendodontic pain"[tiab] OR “pain after root canal treatment"[tiab]) AND (“root canal therapy"[tiab] OR “endodontic treatment"[tiab] OR “pulpitis"[tiab]) AND (“sodium hypochlorite"[MeSH Terms] OR “sodium hypochlorite"[tiab] OR “NaOCl"[tiab]) AND(“cryotherapy"[tiab] OR “intracanal cryotherapy"[tiab] OR “cold"[tiab] OR “precooled"[tiab] OR “pre-cooled"[tiab] OR “temperature-controlled"[tiab] OR “cooled sodium hypochlorite"[tiab] OR “irrigant temperature"[tiab] OR “temperature modification"[tiab]))	8 results
WEB OF SCIENCE	((“postoperative pain” OR “postendodontic pain” OR “pain after root canal treatment”) AND (“root canal therapy” OR “endodontic treatment” OR pulpitis) AND (“sodium hypochlorite” OR “sodium hypochlorite” OR NaOCl) AND (cryotherapy OR “intracanal cryotherapy” OR cold OR precooled OR pre-cooled OR temperature-controlled OR “cooled sodium hypochlorite” OR “irrigant temperature” OR “temperature modification”))	88 results
SCOPUS	((TITLE-ABS (“postoperative pain”) OR TITLE-ABS (“postendodontic pain”) OR TITLE-ABS (“pain after root canal treatment”)) AND (TITLE-ABS (“root canal therapy”) OR TITLE-ABS (“endodontic treatment”) OR TITLE-ABS (pulpitis)) AND (INDEXTERMS (“sodium hypochlorite”) OR TITLE-ABS (“sodium hypochlorite”) OR TITLE-ABS (NaOCl)) AND (TITLE-ABS (cryotherapy) OR TITLE-ABS (“intracanal cryotherapy”) OR TITLE-ABS (cold) OR TITLE-ABS (precooled) OR TITLE-ABS (pre-cooled) OR TITLE-ABS (temperature-controlled) OR TITLE-ABS (“cooled sodium hypochlorite”) OR TITLE-ABS (“irrigant temperature”) OR TITLE-ABS (“temperature modification”)))	5 results
COCHRANE LIBRARY	((“postoperative pain":ti,ab OR “postendodontic pain":ti,ab OR “pain after root canal treatment":ti,ab) AND (“root canal therapy":ti,ab OR “endodontic treatment":ti,ab OR pulpitis:ti,ab) AND ([mh “sodium hypochlorite”] OR “sodium hypochlorite":ti,ab OR NaOCl:ti,ab) AND (cryotherapy:ti,ab OR “intracanal cryotherapy":ti,ab OR cold:ti,ab OR precooled:ti,ab OR pre-cooled:ti,ab OR temperature-controlled:ti,ab OR “cooled sodium hypochlorite":ti,ab OR “irrigant temperature":ti,ab OR “temperature modification":ti,ab))	2 results

(“postoperative pain” OR “postendodontic pain” OR “pain after root canal treatment”) AND (“root canal therapy” OR “endodontic treatment” OR “pulpitis”) AND (“sodium hypochlorite” OR “NaOCl” OR “irrigant temperature” OR “cold sodium hypochlorite” OR “warm sodium hypochlorite”).

### Selection process

Duplicate records were identified and removed after importing all retrieved citations into the Rayyan screening platform (Rayyan Systems Inc., Qatar). The software's automated duplicate detection feature was used to identify repeated records across databases prior to the title and abstract screening stage. Data extraction was performed independently (Maria Anna Geevarghis and Teena S. Dsouza) using a standardized extraction form. Prior calibration was conducted among the reviewers to ensure consistency in data interpretation. The involvement of additional authors facilitated verification of extracted data and resolution of discrepancies, thereby improving the accuracy and reliability of the collected information. After the primary search, 18 duplicate entries were identified amongst which 11 records were removed, 7 were retained, resulting in 92 records eligible for screening. 89 studies unfit according to inclusion criteria were eliminated after the titles and abstracts of these records were evaluated based on the predetermined eligibility criteria. For full-text review, the remaining 3 papers were included. 3 randomized controlled studies met all eligibility requirements and were taken for the qualitative synthesis after careful evaluation.

During screening or full-text assessment any discrepancies between the two primary reviewers (Maria Anna Geevarghis and Teena S. Dsouza) were resolved through discussion, with the participation of a third reviewer (Chitharanjan Shetty) when necessary to achieve agreement.

### Risk of bias summary

Risk of bias in the included randomized controlled trials was assessed using the revised Cochrane Risk of Bias tool (RoB-2) developed by the Cochrane Collaboration (United Kingdom), which evaluates potential bias across multiple methodological domains. Two reviewers independently checked each trial using the five RoB-2 domains: (1) the randomization procedure; (2) deviations from planned interventions; (3) missing outcome data; (4) outcome measurement; and (5) choice of the reported result. Each domain was categorized as low risk, moderate concerns, high risk, no information, or not relevant using the signalling questions and criteria from the RoB-2 recommendations. A study was classified as high risk of bias if at least one domain was rated as high risk or if several domains raised concerns; it was classified as low risk of bias only if all domains were rated as low risk; and it was thought to raise some concerns if at least one domain was rated as such without any domain being rated as high risk. When the two reviewers were unable to reach an agreement through discussion, a third reviewer acted as a mediator.

## Results

After applying filters for the year of publication (2015–2025), randomized controlled trials, and studies relevant to dental and endodontic areas, a thorough electronic search throughout PubMed, Scopus, Web of Science and Cochrane library produced a total of 103 entries. 92 records were filtered by title and abstract after 11 duplicates were removed both automatically and manually. Following title and abstract screening, 89 records were excluded based on predefined eligibility criteria. The primary reasons for exclusion included ineligible patient populations (*n* = 8), use of interventions other than temperature-modified NaOCl (*n* = 35), non-randomized or inappropriate study designs (*n* = 22), retreatment cases (*n* = 3), absence of an appropriate comparator group (*n* = 10), and failure to report postoperative pain as an outcome (*n* = 11). The full texts of the remaining three articles were subsequently retrieved and evaluated in detail. All three studies fulfilled the inclusion criteria and were therefore incorporated into the qualitative synthesis.

**Figure 1:**
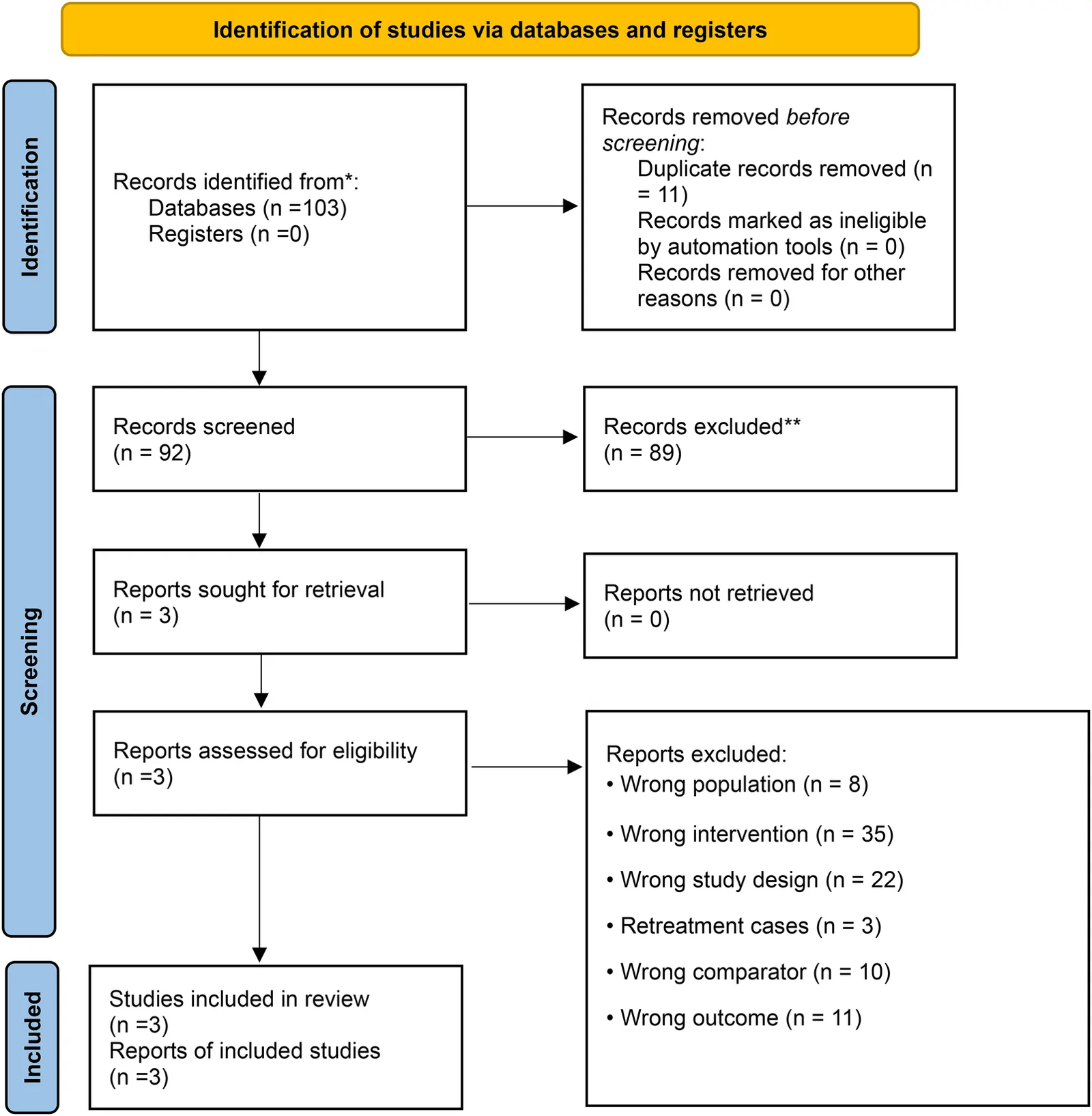
Prisma flow diagram.

The PRISMA flow diagram (Figure 1), outlining the progression from initial search to final study inclusion is depicted below.

The final review included 3 randomized controlled clinical trials, conducted in Turkey, India, and Iran, including 183 participants undertaking non-surgical root canal treatment of mandibular molars diagnosed with irreversible pulpitis or symptomatic apical periodontitis.

A detailed summary of study characteristics is presented in Table 2 (Characteristics of Included Studies).

**Table 2:** Summary of characteristics of included studies.

Author/year	Country	Study design	No. Of participants (n)	Sex & age	Tooth type	Excluded conditions	Temperatures tested	NAOCL concentration
Karataş et al. (9)	Turkey	Randomized Controlled Trial	45 patients (15 per group)	26 females; 19 males; age not specified	Incisors, canine, premolars	-Patients with any systemic disease; previously endodontic treated tooth. -Teeth that prevented proper rubber dam isolation. -The presence of internal or external resorption.	2 °C, 25 °C, 45 °C	5.25% NaOCl
Lalfakawmi et al. (10)	India	Randomized Controlled Trial	66 patients (22 per group)	Sex not reported; Age 18-40 yrs	Mandibular 1st or 2nd molars	-Teeth with well-defined periapical radiolucency. -Teeth with periodontal pocket, calcified canals, aberrant root canal morphology,root resorption, and prior endodontic intervention for the same tooth. -Pregnant or lactating patients and systemic diseases/medications.	2 °C, 25 °C, 66 °C	3% NaOCl
Mokhtari et al. (11)	Iran	Randomized Double-Blind Controlled Trial	72 patients (12 per group)	45-males, 27-females; Age 18–65 yrs	Mandibular molars	– Not achieving lip numbness 15 min after block injection – Pregnancy and lactation – Severe periodontal disease – Teeth that could not be isolated with a rubber dam – Root canal calcification – Teeth with severe irreparable caries – Use of a specific drug or medication	2.5 °C, 22 °C, 40 °C	0.5% & 1% NaOCl

This table outlines information such as authorship, study design, sample size, participant demographics, tooth types treated, exclusion criteria, irrigant temperatures assessed and NaOCl concentration used. A summary of the intervention and protocol followed is depicted in Table 3.

**Table 3:** Summary of protocol/intervention followed in the studies.

Study	Single vs. multi visit	Diagnostic criteria (SIP/SAP/AAP)	Analgesic protocol	Irrigation regimen (volume, needle gauge, type, activation, final rinse)	Instrumentation parameters (working length, apical size, file system)	Obturation/sealer	Attrition/missing data per group
Karataş et al. (9)	Single visit	AAP	No analgesic for 1 month before treatment. Post treatment analgesics can be taken as per need	During preparation:1 mL of 1% NaOCl between three pecking motions of the file. Final irrigation: 5 mL of 17% EDTA followed by 5 mL of 1% NaOCl (2 °C, 25 °C, 45 °C)	Electronic apex locator (Raypex 6; VDW, Munich, Germany), File system Reciproc files (R25, R40 or R50) (VDW, Munich, Germany),	Cold lateral compaction with GP cones and sealer (Sealapex, Kerr Corporation, Orange, CA).	Needle gauge, type of activation, exact WL and apical file size are missing.
Lalfakawmi et al. (10))	Single visit	SIP/ SAP	No analgesic 6 h before treatment. Post treatment: 400 mg Ibuprofen as per need	During preparation, 3% NaOCl was used, and the canals were flushed for 1 min with 2 mL of 17% EDTA. Final irrigation: Four 5-mL syringe NaOCl (Leur-Lock design, 30G side-vented needle) at temperatures 2 °C, 25 °C, 66 °C placed at 1 to 2 mm short of the working length, slowly for about 5 min.	WL: (Root ZX Mini, J Morita Corp., Kyoto, Japan) was confirmed using a #10 Kfile (Mani, Inc., Tochigi, Japan). Mesial canals: TruNatomy file (Dentsply, India) up to apical preparation of size 26 and 0.04 taper. A larger distal canal was prepared up to size 35 and 0.04 taper.	Gutta-percha and sealer (AH Plus, Dentsply Sirona, Mumbai, India) used for obturation.	Type of activation, exact WL and technique of obturation are not specified.
Mokhtari et al. (11)	Single visit	SIP	No analgesics 12 h before treatment. Post treatment analgesic: Ibuprofen is prescribed	During instrumentation, 2 mL of NaOCl between each successive instrumentation (1% & 0.5% solution at 2.5 °C, 22 °C, 40 °C) were used. A 27-G side vented needle (Avapezeshk) up to 2 mm shorter than the working length in an upand down motion was used.and the. Final irrigation:3 mL of 17% ethylenediaminetetraacetic acid (Morvabon) and then with 5 mL of normal saline solution.	WL:Root ZX apex locator (Morita Co.) and radiographically confirmed. Hand instruments to at least #15 K-file (Mani), Denco rotary files (Shenzhen Denco Medical). Apical patency:K-file #10 (Mani), and apical preparation was completed up to the F3 file.	Cold lateral compaction technique with gutta-percha (Meta Biomed Co.) and AH26 sealer (Dentsply Maillefer) were used	Exact WL not specified.

SIP, Symptomatic irreversible pulpitis; SAP, Symptomatic apical periodontitis; AAP, Asymptomatic apical periodontitis; WL, Working Length.

Validated scales (VAS or Heft-Parker VAS) for measuring POP were tested in all studies. NaOCl temperatures tested ranged from 2 °C to 66 °C.Together, these studies provided a reasonable dataset allowing production of the effects of temperature- controlled NaOCl on POP.

Risk of bias was evaluated using the Cochrane RoB-2 tool, examining five domains: randomization process, deviations from intended interventions, missing outcome data, outcome measurement, and selection of reported results. Two reviewers independently assessed each study, with disagreements resolved through discussion.

**Figure 2:**
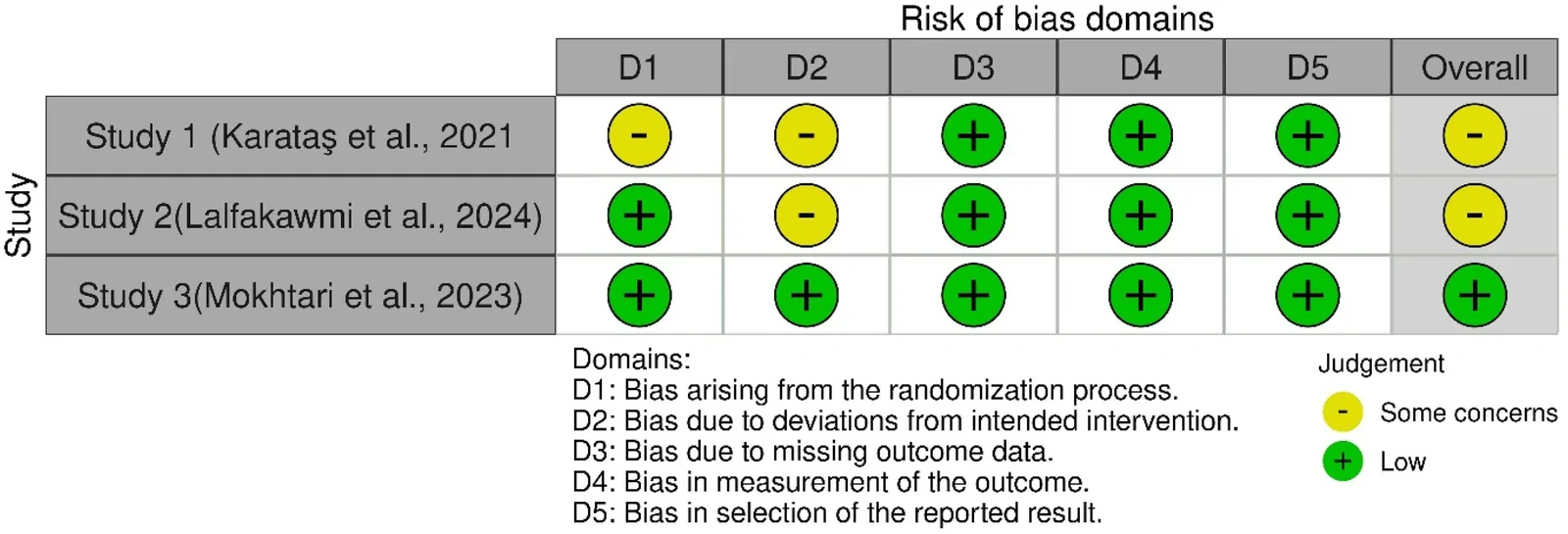
Traffic light plot.

Overall, two studies (9, 10) revealed some concerns, mainly due to incomplete reporting of randomization process (Domain 1) and potential bias arising from deviations from intended interventions (Domain 2). The third study (11) was assessed as having an overall low risk of bias, supported by adequate allocation procedures, appropriate blinding measures, and comprehensive methodological reporting. The RoB assessment is summarized in the traffic-light plot (Figure 2), which displays domain-specific judgments using standardized color indicators (green=low risk; yellow=some concerns). Importantly, none of the included trials was classified as high risk in any domain. The domains most frequently contributing to elevated risk judgments were Domain 1 (randomization process) and Domain 2 (deviations from intended interventions), whereas Domains 3 (missing outcome data), 4 (measurement of outcome), and 5 (selection of reported results) were consistently rated as low risk across studies. Table 4 shows detailed RoB assessment details.

**Table 4:** Detailed Rob assessment chart.

Study	D1 (Randomization process)	D2(Deviations from intended interventions)	D3(Missing outcome data)	D4(Measurement of outcome)	D5(Selection of reported result)	Overall
Study 1 (Karataş et al., 2021)	Some concerns	Some concerns	Low	Low	Low	Some concerns
Study 2(Lalfakawmi et al., 2024)	Low	Some concerns	Low	Low	Low	Some concerns
Study 3(Mokhtari et al., 2023)	Low	Low	Low	Low	Low	Low

### Effect of intervention on postoperative pain

Across the included trials, baseline pain intensity was comparable between intervention groups within each study. In the randomized clinical trial by Karataş et al. (9), postoperative pain on day 1 differed significantly among temperature groups. The mean VAS score (0–100 mm) on day 1 was 6.67 ± 10.72 in the 2 °C group, 16.87 ± 27.60 in the 25 °C group, and 40.0 ± 50.71 in the 45 °C group, with significantly lower pain in the 2 °C group compared with the 45 °C group (*p* < 0.05). No statistically significant difference was observed between the 2 °C and 25 °C groups or between the 25 °C and 45 °C groups. Analgesic intake also differed, with 0, 2, and 5 patients requiring analgesics in the 2 °C, 25 °C, and 45 °C groups, respectively; analgesic consumption was significantly higher in the 45 °C group compared with the 2 °C group (*p* < 0.05) (9).

In the trial conducted by Lalfakawmi et al. (10), preoperative HP-VAS scores (0–170 mm) were similar across groups (control: 68.73; cold: 69.73; warm: 69.41). At follow-up, the cold NaOCl group demonstrated lower HP-VAS values compared with the room-temperature and warm groups at early time points. A statistically significant reduction in pain was observed at 6 h in the cold group compared with the other groups (*p* < 0.05). However, no statistically significant intergroup differences were detected a later time interval. Analgesic intake did not differ significantly among groups (*p* > 0.05), although 36.1%, 31.8%, and 50% of participants in the control, cold, and warm groups, respectively, reported analgesic use (10).

In contrast, Mokhtari et al. (11) evaluated 0.5% and 1% NaOCl at 2.5 °C, 22 °C, and 40 °C in a six-arm double-blind trial using a 0–10 VAS scale. Immediately after treatment, mean VAS scores ranged from 5.0 ± 1.6 to 6.0 ± 1.86 across all temperature and concentration groups. At 3 h, mean values ranged from 6.1 ± 1.6 to 6.33 ± 1.87. Although pain levels changed significantly over time within groups (*p* < 0.001), no statistically significant differences were found between temperature or concentration groups at any time point (*p* > 0.05). Analgesic consumption also showed no significant intergroup differences, although the number of analgesics taken varied significantly over time within each group (*p* < 0.001) (11).

When the findings were considered collectively, two trials (9, 10) reported statistically significant reductions in postoperative pain associated with cold NaOCl at early postoperative intervals, particularly within the first 24 h. In contrast, the six-arm double-blind study (11) did not identify significant differences in postoperative pain intensity or analgesic consumption across varying temperatures or concentrations. No study reported a consistent analgesic advantage for warm NaOCl compared with room temperature. Summary of findings of included studies are mention in Table 5.

**Table 5:** Summary of findings of included studies.

Study	Comparison between (cold/room temperature/warm)	Direction/magnitude of effect	Time points (Preoperatively/immediately/6 h/12 h/day1/day2/day3/day5/day7)	Pain scale used	Key limitations/certainity
Karataş et al. (9)	POP on day 1, 2˚C group reported significantly less than the NaOCl 45˚C group(*p* < 0.05). No statistically significant difference between the NaOCl 2˚C and NaOCl 25˚C groups and between the NaOCl 25˚C and NaOCl 45˚C groups. Postoperative analgesic intake was significantly higher in the NaOCl 45˚C group than in the NaOCl 2˚C group (*p* < 0.05). However, no statistically significant difference between the NaOCl 2˚C and NaOCl 25˚C groups and between the NaOCl 25˚C and NaOCl 45˚C.	Day1 POP, Cold(2 °C): 6.67 ± 10.72, Room(25 °C): 16.87 ± 27.60, Warm(45 °C): 40.0 ± 50.709 The number of patients that needed analgesic was 0, 2 and 5 for the NaOCl 2˚C, NaOCl 25˚C and NaOCl 45˚C groups, respectively.	Day1, day2, day3, day5, day7	VAS(0–100 mm)	Room-temperature NaOCl does not offer additional antibacterial benefits and is associated with higher postoperative pain compared to cold NaOCl when used as a final irrigant in teeth with asymptomatic apical periodontitis.
Lalfakawmi et al. (10)	Lower HP-VAS value in the cold NaOCl group in all the follow-up times compared with the remaining 2 groups. No statistical difference in analgesic intake among the groups (*P* > 0.05).	The mean HP-VAS value was 68.73, 69.73, and 69.41 for the control, cold, and warm groups, respectively, Analgesics in control, cold, and warm NaOCl are 36.1%, 31.8%, and 50% of subjects, respectively.	Preoperatively, day 1, day 2, day 3	HP-VAS (0–170 mm)	Cold NaOCl provided better postoperative analgesia following root canal therapy at 6 h in teeth with SIP and SAP.
Mokhtari et al. (11)	Immediately, 3, 24, 48, 72 h, and 7 days after treatment showed significant changes in VAS pain scores in 2.5 °C, 22 °C, 40 °C for 0.5% & 1% concentration of NaOCl (*p* < .001). The pain decreased immediately and increased 3 h after treatment in all the groups. Hourly, the severity of pain decreased up to 7 days after treatment in all the groups. (*p* = .028). No significant difference in the mean number of analgesics taken by patients in each of the irrigants groups, and the concentration and temperature of NaOCl. However, the number of analgesics taken showed significant differences in each irrigation solution group (*p* < .001). The mean number of analgesics taken by the patients immediately after treatment was the least in all the groups.	Immediately after treatment, the mean VAS scores were 5.5 ± 1.57 (0.5% NaOCl, 22 °C), 6.0 ± 1.71 (0.5%, 40 °C), 5.33 ± 1.3 (0.5%, 2.5 °C), 5.92 ± 1.56 (1%, 22 °C), 6.0 ± 1.86 (1%, 40 °C), and 5.0 ± 1.6 (1%, 2.5 °C); at 3 h, the corresponding values were 6.33 ± 1.23, 6.1 ± 1.6, 6.25 ± 1.14, 6.33 ± 1.44, 6.3 ± 1.71, and 6.33 ± 1.87, respectively. For 0.5% NaOCl, the median (IQR) analgesic intake immediately after treatment was 0 (0–0.75) at 22 °C, 0.5 (0–1) at 40 °C, and 0 (0–0.75) at 2.5 °C.For 1% NaOCl, the median (IQR) analgesic consumption immediately after treatment was 0 (0–1) at 22 °C, 0.5 (0–1) at 40 °C, and 0 (0–0.75) at 2.5 °C; at 3 h, all groups demonstrated a median of 1 with IQRs of (1–1) at 22 °C, (0.25–1) at 40 °C, and (0–1) at 2.5 °C;	Immediately, 3 h, day1, day 2, day 3, day 7	VAS (0–10)	No significant differences among temperatures or concentrations regarding postoperative pain.

HP- VAS, Heft Parker Visual Analog Scale; VAS, Visual Analog Scale.

## Discussion

POP following nonsurgical root canal therapy remains a clinically significant outcome, as it influences patient comfort, treatment satisfaction, and the incidence of unscheduled emergency visits. The etiology of this pain is multifactorial, involving residual microbial infection, mechanical insult to periapical tissues, extrusion of irrigants or debris, and host inflammatory responses. Within this context, the characteristics of the irrigating solution play a critical role in modulating postoperative symptoms. NaOCl, the irrigant of choice in endodontic practice, is widely valued for its antimicrobial efficacy and tissue-dissolving capacity; however, its cytotoxic nature may irritate periapical tissues and thereby contribute to POP (1, 7). Because the biological behavior of NaOCl is influenced by temperature, adjusting the irrigant's thermal profile has been proposed as a means to alter both its chemical activity and its effects on periradicular tissues. The use of warmed NaOCl is theoretically supported by evidence showing that higher temperatures increase the availability of active chlorine, thereby improving organic tissue dissolution and enhancing antimicrobial performance (8, 12).

Previous research has shown that heating NaOCl to temperatures in the range of 45 °C to 60 °C markedly enhances its ability to dissolve organic tissue and improves its antibacterial action. These effects are thought to promote more effective canal disinfection, potentially limiting the presence of residual irritants that could trigger POP. In contrast, the use of cooled NaOCl is based on the biological principles of cryotherapy, whereby lower temperatures may attenuate postoperative inflammation by decreasing vasodilation, inflammatory mediator release, neuropeptide activity, and overall metabolic activity within the periapical tissues (13, 14). These anti-inflammatory properties have been linked to a reduction in POP when cold saline or cold NaOCl is employed as the final irrigant after canal instrumentation. Nevertheless, the outcomes of the three randomized controlled trials included in the present review suggest that the association between irrigant temperature and POP is variable and not consistently observed across studies.

Two trials—Karataş et al. (9) and Lalfakawmi et al. (10). Both studies documented a consistent decrease in early POP when NaOCl was cooled to approximately 2 °C. Patients in the cold-irrigation groups exhibited significantly lower pain scores at 6- and 24-hour intervals compared with those receiving warm or room-temperature irrigants. These observations are in agreement with earlier intracanal cryotherapy studies, which reported reduced postoperative discomfort following the use of cold saline after canal instrumentation (13, 14). The proposed explanation is based on well-established cryotherapy mechanisms, including a reduction in intraosseous blood flow and suppression of inflammatory mediators such as prostaglandins and substance P. These physiological effects have been consistently demonstrated in musculoskeletal injuries and postoperative surgical settings and are thought to account for the observed reduction in pain (15).

In contrast, Mokhtari et al. (11) The study did not detect any meaningful differences between the two NaOCl concentrations when used at 2.5 °C, 22 °C, or 40 °C. This variation in findings may be attributed to several methodological factors. First, the use of rigorous double-blinding likely minimized expectancy-related bias in patient-reported pain outcomes. Second, the included teeth presented with varying degrees of preoperative inflammation, which may have influenced their responsiveness to temperature modulation. Finally, differences in irrigant volume, duration of contact, and method of delivery—compared with earlier studies—could have altered NaOCl penetration and the extent of temperature change at the periapical tissues (6, 16). These inconsistencies highlight the challenge of translating temperature-related effects into predictable clinical benefits. Although heating NaOCl increases its chemical reactivity, this enhanced activity does not necessarily lead to improved pain outcomes. In teeth with vital or inflamed pulps, where nociceptor activity is already heightened, warm irrigants may improve debridement efficiency but can simultaneously increase the likelihood of periapical irritation. Findings from studies on irrigant extrusion indirectly support this concern (7). Accordingly, although heating NaOCl may improve canal cleanliness, it does not necessarily offer an advantage in minimizing short-term postoperative discomfort. The clinical condition of the tooth also plays a critical role. Teeth diagnosed with symptomatic irreversible pulpitis typically exhibit heightened inflammatory responses and increased pain sensitivity, circumstances in which cryotherapy may be physiologically more advantageous than enhanced chemical dissolution alone. In contrast, in necrotic cases—where bacterial burden is greater and tissue sensitivity is reduced—warmed irrigants may provide more meaningful antimicrobial benefits without a comparable risk of pain exacerbation (5, 12).

Future research should place greater emphasis on differences in pulpal status, as this factor may play a key role in explaining the variability observed across clinical trials. Meaningful comparisons are further complicated by methodological inconsistencies between studies. Variations in irrigant volume, methods used to maintain temperature, final irrigation protocols, diagnostic criteria, and pain assessment tools can mask true temperature-related effects. This concern is reinforced by risk-of-bias assessments, which indicate that inadequate reporting of randomization procedures and the absence of blinding in two studies increase their susceptibility to subjective reporting bias—an especially important limitation when evaluating pain outcomes. Given its inherently subjective nature, pain perception is strongly influenced by factors such as patient anxiety, expectations, and cultural context (17).

Methodological rigor, particularly with respect to allocation concealment and adequate blinding, is essential for generating reliable and interpretable results. Overall, the existing evidence suggests that although warming NaOCl enhances its tissue-dissolving capacity, it does not consistently confer analgesic benefits. In contrast, the use of cold sod NaOCl appears to be associated with a short-term reduction in POP, most notably within the first 24 h after treatment. The inconsistent findings across trials indicate that irrigant temperature alone is unlikely to be an independent determinant of POP. Instead, its effects likely depend on interactions with factors such as pulpal status, irrigant concentration, method of delivery, baseline inflammatory activity, and individual pain sensitivity. Nevertheless, given its biological rationale, ease of implementation, and minimal cost, cold NaOCl irrigation may represent a practical adjunct for managing acute postoperative discomfort.

### Strengths and limitations

This review has several methodological strengths, including a comprehensive search across multiple databases, strict compliance with PRISMA guidelines, prior registration in PROSPERO, and independent study selection and data extraction conducted by multiple reviewers. The inclusion of only randomized controlled trials ensured that the highest level of available evidence was synthesized. Moreover, the detailed extraction of irrigation parameters specific to temperature and concentration enhanced the clinical relevance and interpretability of the findings.

Nevertheless, certain limitations must be acknowledged. The inclusion of only three randomized controlled trials restricts the generalizability of the conclusions. In addition, clinical and methodological heterogeneity arose from variations in NaOCl concentration, irrigant volume, instrumentation systems, obturation techniques, and pain assessment methods. A moderate overall risk of bias was observed in two studies, primarily driven by concerns related to the randomization process and potential deviations from intended interventions, rather than deficiencies in outcome measurement or assessor blinding. Formal quantitative standardization was not performed because variations in pain measurement scales, follow-up intervals, intervention protocols, and study design introduced substantial heterogeneity, limiting the validity of pooled or rescaled comparisons. Finally, differences in pulpal and periapical diagnoses across studies may have influenced pain outcomes and contributed to inter-study variability.

### Clinical implications

The findings of this review carry meaningful clinical implications for endodontic practice. In cases of symptomatic irreversible pulpitis or symptomatic apical periodontitis, the use of cold NaOCl maintained at approximately 2–4 °C may represent a simple and cost-effective approach to reducing postoperative discomfort. Given that POP remains a common contributor to patient dissatisfaction and unscheduled emergency visits, intracanal cryotherapy offers a pragmatic adjunct that can be readily implemented without additional equipment beyond standard refrigeration.

At the same time, warm NaOCl continues to have a role in specific clinical scenarios, particularly in necrotic canals, where enhanced tissue dissolution and antimicrobial activity are desirable. The present findings do not contraindicate the use of heated irrigants; rather, they suggest that their application should be judicious, especially when managing inflamed vital pulps in which the risk of POP is greater.

Until more definitive evidence becomes available from future clinical trials, clinicians should individualize the choice of irrigant temperature, taking into account pulpal status, procedural complexity, and patient comfort requirements, in light of the inconsistent findings reported in the current literature.

## Conclusion

This review evaluated the effect of temperature-modified NaOCl irrigation on POP following nonsurgical root canal therapy. Across the three included randomized clinical trials, the use of cold NaOCl demonstrated a consistent tendency toward reduced early POP, particularly within the first 24 h, when compared with room-temperature or heated irrigants. However, the strength of these findings is constrained by methodological heterogeneity and variability in statistical significance among the studies. Although intracanal cryotherapy appears to be a promising adjunct for POP control, further well-designed, standardized clinical trials are required to confirm its efficacy and to inform evidence-based clinical recommendations.

## Data Availability

The original contributions presented in the study are included in the article/Supplementary Material, further inquiries can be directed to the corresponding author.
